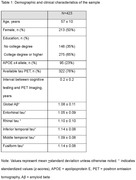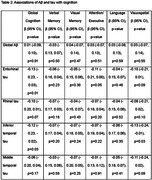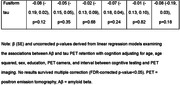# Examining early cerebral amyloid beta and tau accumulation in association with cognition in a predominantly middle‐aged cohort

**DOI:** 10.1002/alz.087431

**Published:** 2025-01-09

**Authors:** Mitzi M. Gonzales, Adrienne O’Donnell, Saptaparni Ghosh, Emma G Thibault, Jeremy A. Tanner, Claudia L Satizabal, Charles Decarli, Georges El Fakhri, Keith A Johnson, Alexa S Beiser, Sudha Seshadri, Matthew P. Pase

**Affiliations:** ^1^ Glenn Biggs Institute for Alzheimer’s & Neurodegenerative Diseases, University of Texas Health Science Center, San Antonio, TX USA; ^2^ Cedars‐Sinai Medical Center, Los Angeles, CA USA; ^3^ Boston University, Boston, MA USA; ^4^ The Framingham Heart Study, Framingham, MA USA; ^5^ Boston University Chobanian & Avedisian School of Medicine, Boston, MA USA; ^6^ Gordon Center for Medical Imaging, Massachusetts General Hospital, Boston, MA USA; ^7^ Framingham Heart Study, NHLBI, Framingham, MA USA; ^8^ Glenn Biggs Institute for Alzheimer’s & Neurodegenerative Diseases, University of Texas Health Science Center at San Antonio, San Antonio, TX USA; ^9^ University of California, Davis, CA USA; ^10^ Gordon Center for Medical Imaging, Massachusetts General Hospital, Harvard Medical School, Boston, MA USA; ^11^ Gordon Center for Medical Imaging, Department of Radiology, Division of Molecular Imaging and Nuclear Medicine, Massachusetts General Hospital, Harvard Medical School, Boston, MA USA; ^12^ Boston University School of Medicine, Boston, MA USA; ^13^ Glenn Biggs Institute for Alzheimer’s & Neurodegenerative Diseases, University of Texas Health Sciences Center at San Antonio, San Antonio, TX USA; ^14^ Harvard T.H. Chan School of Public Health, Harvard University, Boston, MA USA; ^15^ Turner Institute for Brain and Mental Health & School of Psychological Sciences, Monash University, Clayton, VIC Australia

## Abstract

**Background:**

In older adults, greater amyloid (Aβ) and tau positron emission tomography (PET) binding is associated with cognitive decline and dementia. However, the association of early amyloid and tau PET accumulation with cognition at midlife remains unclear. The goal of the current study was to evaluate the associations of Aβ and tau PET with cognition in a predominately middle‐aged community‐based cohort, as well as to examine the factors that may modify these associations.

**Method:**

Participants from the observational Framingham Heart Study completed cognitive assessments and underwent ^11^C‐Pittsburgh Compound B (PiB) Aβ and ^18^F‐Flortaucipir tau PET imaging. Associations of standardized quantitative measures of Aβ (global composite) and tau (five early regions of interest) PET retention with cognition were evaluated using linear regression models. Interactions with age, apolipoprotein ε4 status, and education were examined and stratified analyses were performed where interactions were significant. All statistical tests were two‐sided and were FDR‐corrected to account for multiple comparisons

**Result:**

The sample included 423 participants (mean age 57±10 years, 50% female, Table 1). There were no significant associations between Aβ or tau and cognition in the overall sample after correcting for multiple comparisons (Table 2). Stratified analyses indicated a significant association between higher fusiform gyrus tau and poorer visuospatial function in those younger than 55 years (b=‐0.27, 95% CI ‐0.46 to ‐0.07, p=0.007) and associations between higher inferior and middle temporal tau with poorer visuospatial function in those without a college degree (inferior temporal b=‐0.25, 95% CI ‐0.02 to 0.41, p=0.01, middle temporal b=‐0.25, 95% CI ‐0.22 to 0.0, p=0.02).

**Conclusion:**

The continuous range of amyloid beta and tau accumulation at midlife was not cross‐sectionally associated with cognition. However, the findings suggest that higher tau accumulation at younger ages or in the context of lower cognitive reserve may increase vulnerability for poorer cognition as early as midlife.